# Supplementary material for: A Hypoxia-Inducible Factor Stabilizer Improves Hematopoiesis and Iron Metabolism Early after Administration to Treat Anemia in Hemodialysis Patients
Source: Int J Mol Sci. 2020 Sep 28;21(19):7153. doi: 10.3390/ijms21197153 (PMC7583824; doi:10.3390/ijms21197153)
Supplement: Supplementary file 1 [file ijms-21-07153-s001.pdf]

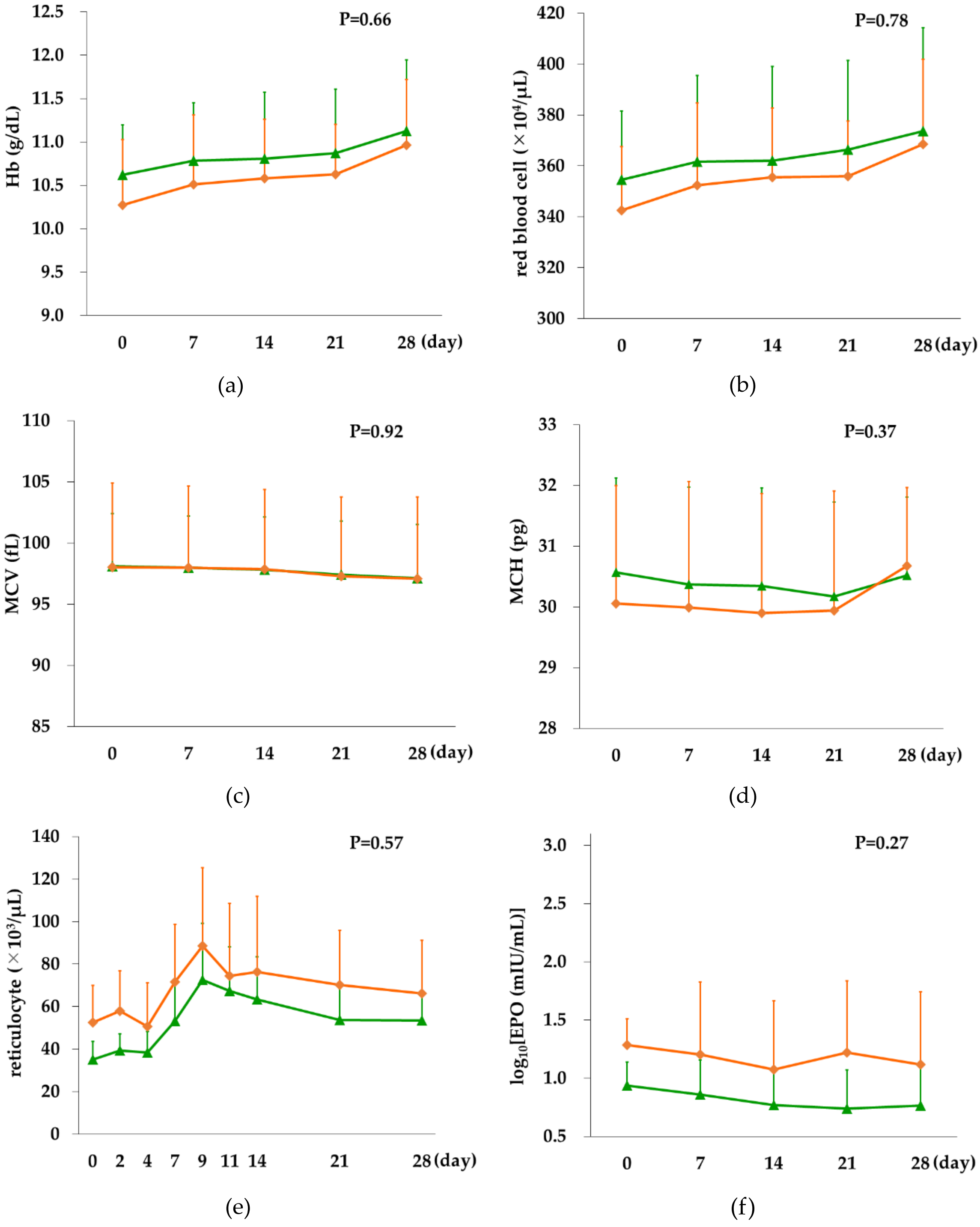

Figure S1. Comparison in erythrocyte indices and EPO concentration between the patients received Rox 70mg and those received Rox 100mg

a) Hb, b) RBC count, c) MCV, d) MCH, e) Ret count, f) Log_{10}(EPO concentration). All variables showed no significant difference between the groups. Two-way repeated measures analysis of variance was performed. The data are represented as mean ± SD.

(—▲— Rox 70mg, —◆— Rox 100mg)

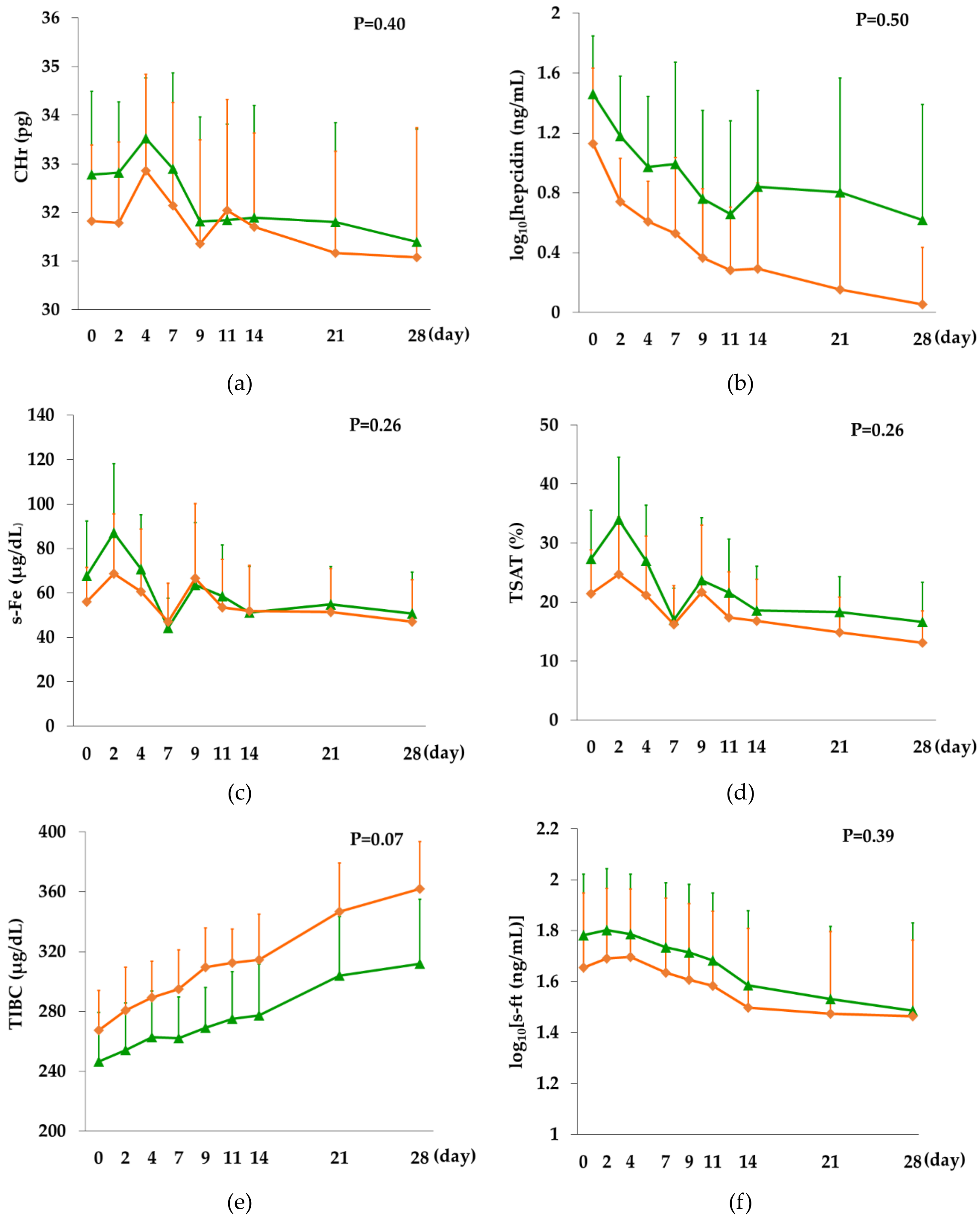

Figure S2. Comparison in CHr, hepcidin levels and iron-related indices between the patients received Rox 70mg and those received Rox 100mg
a) CHr, b) Log$_{10}$(hepcidin), c) s-Fe, d) TSAT, e) TIBC, f) Log$_{10}$(s-ft). All variables showed no significant difference between the groups. Two-way repeated measures analysis of variance was performed. The data are represented as mean ± SD. (▲ Rox 70mg, ◆ Rox 100mg)
